# Expression of ANGPTL2 and its impact on papillary thyroid cancer

**DOI:** 10.1186/s12935-019-0908-9

**Published:** 2019-07-30

**Authors:** Longyan Yang, Rongxin Sun, Yan Wang, Ying Fu, Yuanyuan Zhang, Zhaohui Zheng, Zhili Ji, Dong Zhao

**Affiliations:** 1 Beijing Key Laboratory of Diabetes Prevention and Research, Department of Endocrinology, Luhe Hospital, Capital Medical University, Beijing, 101149 China; 2 Department of General Surgery, Luhe Hospital, Capital Medical University, Beijing, 101149 China

**Keywords:** Papillary thyroid carcinoma, Angiopoietin-like 2, Proliferation, Metastasis, Prognosis

## Abstract

**Background:**

Although the most thyroid carcinoma patients have good prognosis, around 20% of papillary thyroid carcinoma (PTC) patients have a high rate of metastasis and recurrence after routine treatment, which causes high lethality with these patients. Tumor proliferation, metastasis, and invasion are important predictors of PTC invasiveness and are key factors in cancer-related death. Angiopoietin-like 2 (ANGPTL2), a secreted protein which belongs to the angiopoietin (ANGPTL) family, was reported to be involved in the regulation of several different type of cancer cell proliferation and metastasis. However, whether ANGPTL2 plays a role in the progression of PTC, particularly in metastasis and recurrence of PTC, remains unclear. Hence, the purpose of this study was to evaluate the level of ANGPTL2 in PTC and normal thyroid, as well as para-cancerous tissue. Furthermore, the impact of ANGPTL2 on PTC cell proliferation, metastasis, recurrence and invasion was assessed to investigate the possibility whether ANGPTL2 may become a novel target for PTC therapy and cancer prognosis.

**Materials and methods:**

The level of ANGPTL2 in PTC and para-cancerous tissue was assessed by immunohistochemistry. The biological effect of ANGPTL2 on thyroid cancer cell proliferation and metastasis was investigated by the Cell Counting Kit-8 (CCK8) assay, cell scratch test, and transwell assay. Correlations of ANGPTL2 expression levels with proliferation, migration, and metastasis of thyroid cancer were assessed with the TCGA data set and analyzed by gene set enrichment analysis. Receiver operating characteristic analysis was used to evaluate the utility of ANGPTL2 as a biomarker for prediction of thyroid cancer. Survival analysis was performed using the thyroid cancer database in K–M Plotter to detect correlations between survival time and ANGPTL2 levels.

**Results:**

Current study revealed that: (1) ANGPTL2 was highly expressed in thyroid cancer in comparison with adjacent normal thyroid tissue; (2) ANGPTL2 expression was increased with thyroid tumor progression; (3) ANGPTL2 increased proliferation of thyroid cancer cells; (4) ANGPTL2 promoted migration and invasion of thyroid cancer cells; (5) high level of ANGPTL2 in thyroid cancer patients were significantly associated with a poor prognosis. The patients showed a higher metastasis and recurrence rate.

**Conclusion:**

ANGPTL2 promoted and enhanced proliferation, metastasis, and invasion of thyroid cancer cells. ANGPTL2 may be considered as a potential biomarker for diagnosis and prognosis of thyroid cancer patients. Further evaluation needs to be done to analyze the possibility of taking ANGPTL2 as a prognostic marker and therapeutic target for papillary thyroid cancer.

**Electronic supplementary material:**

The online version of this article (10.1186/s12935-019-0908-9) contains supplementary material, which is available to authorized users.

## Background

Thyroid cancer is the most common endocrinal malignant tumor, with an annually increased global incidence [1, 2]. While papillary thyroid carcinoma (PTC) accounts for 80% of all thyroid cancers [3]. The most patients with thyroid cancer have a relatively good prognosis comparing to other type of cancers [4]. However, there is a high rate of metastasis and recurrence after routine treatment in certain part of PTC patients [5, 6]. Approximately 5% to 20% of PTC patients have metastasis to the regional lymph nodes, associated with increased mortality [7, 8]. Although, huge amount of research work have illustrated that many signaling pathways and genes (such as RAS, MAPK and PI3K/Akt signaling pathways; as well as gene BRAF, RAS, RAF, MEK and ERK, etc.), were associated with the occurrence, development, and metastasis of thyroid cancer [9–12]. However, the exact mechanism, which makes such difference, is not clear yet and be unsolved puzzle since long time. Therefore, it would be essential and very important to find out the reason, which causes this special phenomenon and discover the molecular basis behind. Of cause, the finding will great help to better understand the mechanistic basis for PTC proliferation, migration, invasion, and metastasis. Such an understanding would provide insight into potential therapeutic treatments.

Angiopoietin-like protein 2 (ANGPTL2) belongs to angiopoietin-like protein family. The molecule function has been revealed to be involved in angiogenesis regulation, glucose and lipid metabolism. ANGPTL2 is abundantly secreted by adipose tissue (visceral fat is a primary source) [13]. Research evidence suggested that serum level of ANGPTl2 correlated with the degree of adiposity, insulin resistance, and inflammation [14]. More interestingly, recent studies have reported high expression of ANGPTL2 in various malignant tumors including; non-small cell lung cancer [15, 16], colorectal cancer [17], prostate cancer [18], and gastric cancer [19]. These studies suggested that ANGPTL2 enhanced likely proliferation, metastasis, and invasion in these tumors. A recent study on obesity by Haiyoung Son and his colleagues demonstrated that obesity might act as a risk factor associated positively with thyroid cancer [20]. However, so far no evidence clarify the effect of ANGPTL2 on thyroid cancer. Hence, this study is aiming to investigate the difference on expression levels of ANGPTL2 in PTC, normal thyroid, and para-cancerous tissue, meanwhile to assess the effect of ANGPTL2 on thyroid cancer cell proliferation, metastasis, and invasion by biochemistry and bioinformatic analysis with both cell culture study and clinical research information. The result of this study will help to improve the thyroid cancer therapies and prognosis.

## Materials and methods

### Thyroid cancer patient sample and data set collection

We obtained 36 pairs of papillary thyroid carcinoma and adjacent normal thyroid tissue from thyroidectomies conducted at the Beijing Luhe Hospital between 2016 and 2017. In this study, patients referred to Luhe Hospital Capital Medical University (in Beijing, China), for near-total or total thyroidectomy, from January 2016 to January 2017, were initially enrolled. Tumor tissues, adjacent normal tissues from the same case of 36 patients with PTC (29 females and 7 males) were selected based on postoperative pathological reports. All confirmed thyroid samples were immobilized by formalin and embedded in paraffin. The basic and pathological characteristics of PTC patients were extracted from medical records. Tumor staging was determined using the 7th edition of the American Joint Committee on Cancer Tumor-Node-Metastasis (AJCC-TNM) staging system. The research was approved by the Research Ethics Board of Luhe Hospital Capital Medical University and was carried out according to the World Medical Association Declaration of Helsinki. All patients included in the protocol signed a declaration of informed consent.

### The Cancer Genome Atlas (TCGA) data

In addition, mRNA expression data (RNA Seq v2) and clinical information for patients in The Cancer Genome Atlas thyroid cancer data set were downloaded from https://www.synapse.org and cBioPortal database (http://www.cbioportal.org), respectively, and used for analysis of differential mRNA expression and clinical prognosis.

### Cell culture and transfection

Cells were purchased from the National Infrastructure of Cell Line Resource (Beijing, China). Papillary thyroid cancer cell lines TPC-1 and BCPAP were cultured for 48 h in Roswell Park Memorial Institute (RPMI) 1640 medium (Gibco, Cleveland, TN, USA), with 10% fetal bovine serum (FBS) (Gibco, Cleveland, TN) and 1% penicillin/streptomycin in a 37 °C/5% CO_2_ incubator. Cell transfection was performed with Lipofectamine 2000 (Thermo Fisher-Invitrogen, Carlsbad, CA, USA) according to manufacturer’s instructions. Small interfering RNA (siRNA-ANGPTL2: sc-72351) duplexes were purchased from Santa Cruz (Dallas, TX, USA). The ANGPTL2 siRNA was a pool of 3 target-specific 19–25 nt siRNAs designed to knock down ANGPTL2 gene expression. Full-length cDNA encoding human ANGPTL2 were cloned into the vector GV366 plasmid (Shanghai Genechem Co., Ltd) with a HA-tag. Cultured cells were harvested 48 h after transfection in sodium dodecyl sulfate (SDS) sample buffer and were analyzed by western blot to investigate the ANGPTL2 expression change. ANGPTL2 antibody (ab199133) was purchased from Abcam. Polyclonal rabbit anti-HA (#561) antibody was purchased from MBL (Nagoya, Japan). Anti-GAPDH antibody was obtained from ZSGB-BIO (Beijing, China). Otherwise, cells were collected at required time point for different treatment base on the requirement of different analysis methods.

### Proliferation assay

A Cell Counting Kit-8 (CCK8) assay was used to assess cell proliferation rate. Cells were seeded at a density of 2000 cells/well into 96-well plates after 24 h transfection. The cells attached to the plates after 4 h’ incubation and were considered as 0 time point. The viable cells assessed by CCK8 assay (Dojindo, Kumamoto, Japan) using an Enspire microplate reader (Perkin Elmer, Waltham, MA, USA) at 450 nm with 0, 24, 48, 72, 96, and 120 h cell collection.

### Wound-healing assay

A total of 3 × 10^5^ cells were seeded into 6-well plates for 24 h incubation, followed by transfection. The transfected cells were incubated for another 48 h to allow the cells reaching 90% confluence. Cell monolayer was carefully scratched using a sterile 200 μL pipette tip through the center of wells. Detached cells were removed by washing twice with 1 × PBS. Cells were maintained in fresh medium for the indicated time periods. Images of the wound were recorded with a phase contrast microscope. Wound surface widths were measured using NIH Image J 1.62 software. The wound-healing assay was repeated four times.

### Transwell invasion assay

Cell invasion assay was performed using modified Boyden chambers in 24-well plates with 8 μm pore inserts (BD, Biosciences) coated with 1 mg/mL Matrigel. In the upper chamber, 5 × 10^4^ cells were plated in 100 μL of starving medium after 24 h transfection. The lower chamber contained 600 μL of complete medium. After 24 h of incubation at 37 °C, invaded cells were fixed with 4% paraformaldehyde and were stained with 0.5% Crystal Violet. For each experiment, the numbers of cells in five separate light microscopy fields were counted at 200× magnification. The transwell invasion assay was repeated three times.

### Immunohistochemistry

For IHC staining, in order to get better antigen retrieval, deparaffinizing and rehydrating pretreatment of the samples are required. After blocking, samples were then incubated with primary antibody ANGPTL2 (ab199133), followed by washing, secondary antibody incubation and final washing steps according to the standard antibody staining protocol [21]. The staining score for primary tumor and adjacent normal tissue was recorded. The percentage of cells with positive staining and the staining intensity (0, negative; 1+, weak; 2+, moderate; and 3+, strong) were recorded. An H-score was calculated using the following formula: H-SCORE = ∑(PI × I) = (percentage of cells with weak intensity × 1) + (percentage of cells with moderate intensity × 2) + (percentage of cells with strong intensity × 3). A maximum H-score would be 300, corresponding to 100% of the cells exhibiting strong intensity.

### Gene set enrichment analysis

The expression level of ANGPTL2 was assessed by Gene Set Enrichment Analysis. The gene sets were obtained from the Molecular Signatures Database of the Broad Institute (http://software.broadinstitute.org/gsea/msigdb). Tests were performed using default settings, with permutation number set at 1000. A false discovery rate of < 0.25 was considered statistically significant.

### Statistical analysis

Statistical analysis was performed using SPSS 18.0 (SPSS Inc., Chicago, IL, USA). Results are expressed as mean ± SD. Two-tailed unpaired Student’s *t*-test was used to determine statistical significance. Statistical significance was accepted for p < 0.05.

## Results

### ANGPTL2 expression in thyroid cancer and adjacent normal thyroid tissue cells

**Fig. 1:**
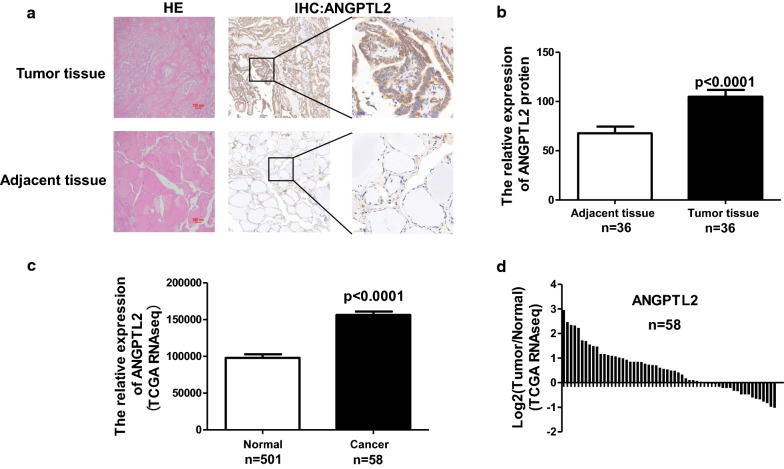
ANGPTL2 is highly expressed in thyroid cancer compared to adjacent normal thyroid tissue. **a** Representative images of ANGPTL2 immunohistochemical staining of thyroid cancer and its adjacent tissue. Scalebars: 200 μm and 50 μm. H&E, hematoxylin and eosin. **b** ANGPTL2 protein levels of 36 human thyroid cancers and adjacent tissues analyzed by immunohistochemistry, p < 0.0001. **c** ANGPTL2 expression in thyroid cancer (N = 501) and normal thyroid tissue (N = 58), p < 0.0001. Data were obtained from the TCGA dataset. **d** ANGPTL2 expression level was upregulated in thyroid cancer tissue comparing to correspond adjacent normal tissue from the TCGA dataset

So far, no specific analysis has been done for the expression and function of ANGPTL2 on thyroid cancer. Therefore, 36 PTC patients based on postoperative pathological reports (29 females and 7 males) were collected. The clinicopathologic characteristics of patients with thyroid carcinoma were showed in Table 1. The examples of immunohistochemical (IHC) staining for ANGPTL2 on human PTC and adjacent noncancerous tissue were displayed in Fig. 1a. The expression level of ANGPTL2 in all 36 cases of human PTC and adjacent noncancerous tissue were measured using the H-score system (see “Material and methods”) based on the IHC staining results. According to the IHC scoring system, the mean ± SD ANGPTL2 IHC score for cancer was 105.0 ± 6.8, and the score for noncancerous tissue was 67.8 ± 6.7. The data revealed that ANGPTL2 expression was significantly upregulated in PTC tissue compared to adjacent noncancerous tissue (p < 0.0001, Fig. 1b).

**Table 1:** Clinicopathologic characteristics of patients with thyroid carcinoma

Variable	No.
No. of patients	36
Age (mean ± SD) (in years)	49.36 ± 14.93
Gender	
Male	7
Female	29
Size (cm)	
≦ 5	29
> 5	7
T stage	
T1	27
> T1	9
Lymph node metastasis	
N0	23
N1	13
Stage	
I	20
> I	16

Next, we would like to check the increase in protein level was based on more translation or on enhanced protein stability. Thus, ANGPTL2 mRNA level from TCGA’s RNA-seq data in thyroid cancer and adjacent tissue was analyzed. Results showed that ANGPTL2 mRNA level was significantly upregulated in thyroid cancer cell comparing to adjacent non-tumor thyroid tissue cell (p < 0.0001, Fig. 1c, d). Taking together, these data suggested that both mRNA and protein level of ANGPTL2 were increased in PTC tissue comparing to adjacent noncancerous tissue.

### ANGPTL2 expression and thyroid tumor stage

**Fig. 2:**
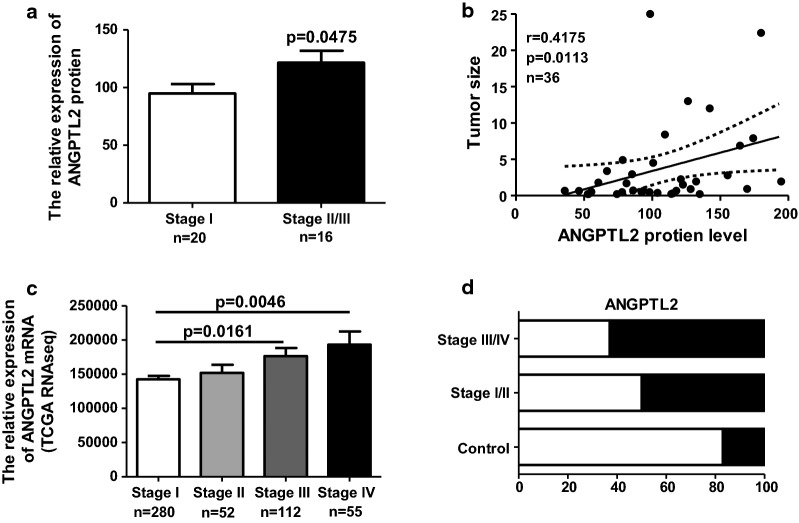
ANGPTL2 expression increases with thyroid tumor progression. **a** ANGPTL2 protein levels were increased in advanced tumor stage, p < 0.05. **b** ANGPTL2 protein levels positively correlated with tumor volume, p < 0.05 r = 0.4175. **c**, **d** ANGPTL2 mRNA expression levels increased with an increase in thyroid tumor stage. Data were obtained from the TCGA dataset

To explore the clinical relevance of ANGPTL2 in thyroid cancer, the relative expression level of ANGPTL2 protein was analyzed using H-score system for PTC tissue in different clinical stages. The patients of stage 2 and 3 were combined in one group due to the small number in each stage. The result showed that ANGPTL2 levels were increased in the advanced stage of PTC tumor (Fig. 2a). Next, we checked the ANGPTL2 protein level in PTC tissue with different tumor size. The correlation between ANGPTL2 protein level and tumor size was evaluated. Results showed that ANGPTL2 protein was positively correlated with PTC tumor volume (Fig. 2b). Similarly in TCGA’s data, with increasing of thyroid tumor severity stage, ANGPTL2 mRNA expression levels increased (Fig. 2c). The ANGPTL2 mRNA in normal thyroid tissue and thyroid cancer tissue with different severity stages (I, II, III, IV) were divided into ANGPTL2 high/low expression groups according to median respectively. The ratio of case number with ANGPTL2 high/low mRNA level from different thyroid cancer stages was compared. The ratio of the cases with ANGPTL2 mRNA level high expression was increased along with the development of PTC tumor (Fig. 2d). These data indicated that the advanced tumor stage and tumor growth might be associated with ANGPTL2 expression.

### ANGPTL2 promotes cell proliferation, migration and invasion of thyroid cancer cells

Tumor cell proliferation and metastasis are important indicators of malignant phenotype. We have found that ANGPTL2 protein level was positively correlated with tumor volume and ANGPTL2 mRNA or protein level were increased along with cancer severity stage, so the effects of ANGPTL2 expression on thyroid cancer cell proliferation, migration and invasion were investigated.

**Fig. 3:**
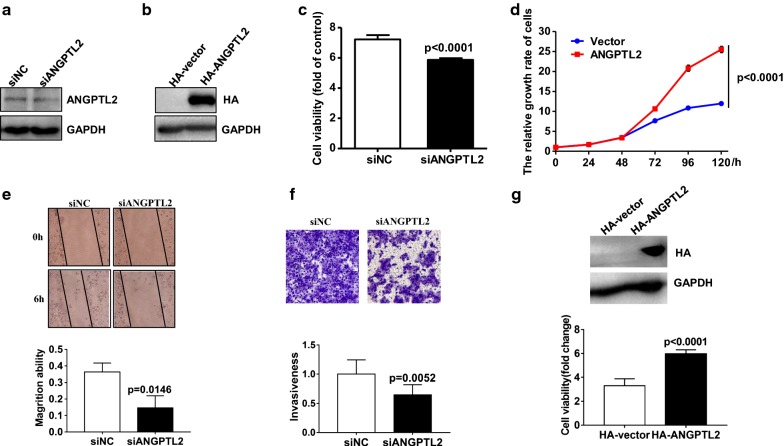
ANGPTL2 increases cell proliferation and migration/invasion on thyroid cancer cell. **a** ANGPTL2 knockdown by siRNA in TPC-1 cells was confirmed by immunoblotting analysis; **b** Overexpression of ANGPTL2 in TPC-1 cells verified by immunoblotting analysis; GAPDH was used as a loading control. **c** ANGPTL2 knockdown inhibited cell proliferation of TPC-1 cells. Growth of TPC-1 cells was assessed by measuring absorbance at 450 nm. Values are expressed as fold change compared with cells at 0 h, (p < 0.05). **d** ANGPTL2 overexpression significantly enhanced cell proliferation of TPC-1 cells. **e** ANGPTL2 knockdown inhibited cell migration by the wound-healing assay. The statistical result was showed on the bottom. **f** ANGPTL2 knockdown restrained cell invasion. The Boyden chambers invasion assay was used. We counted the numbers of cells in five separate light microscopy fields at ×200 magnification (n = 3). Then we putted the statistic result of cell number per well after washing out the crystal violet to the bottom of figure (**f**). The statistical result was showed on the bottom. **g** ANGPTL2 overexpression significantly promoted cell proliferation in BCPAP cells. Overexpression of ANGPTL2 in BCPAP cells were verified by western blot (top). Overexpression of ANGPTL2 promoted the proliferation in BCPAP cells (bottom)

In order to understand the role of ANGPTL2 in thyroid cancer cell proliferation, TPC-1 thyroid cancer cells were transfected with an expression construct carrying either cDNA ANGPTL2 or siRNA-ANGPTL2 respectively, which results in either overexpression or down regulation of ANGPTL2 in TPC-1. After 48 h’ transfection, the outcome level of ANGPTL2 was confirmed by western blotting (Fig. 3a, b). Cell viability was measured, and the ANGPTL2 down-expressed cells were cultured in 96-well plates and stained with CCK8 at 72 h. As the result shown, ANGPTL2 knock-down in TPC-1 cells decreased cell proliferation (Fig. 3c). In contrast, cell proliferation increased in cells over expressing ANGPTL2 as judged by the CCK8 viability assays (Fig. 3d). Putting together, these data demonstrated ANGPTL2 expression to promote thyroid cancer cell proliferation.

To investigate the role of ANGPTL2 in thyroid cancer metastasis, we evaluated migration of thyroid cancer cells by scratch. The assay results showed that the migration areas were smaller in ANGPTL2 knockdown cells comparing to siNC treatment (Fig. 3e). Similar results were obtained by Transwell invasion assay. As displayed with staining of cells in matrigel-coated Boyden chambers, Knocking down ANGPTL2 expression, the TCP-1 cellular invasion was inhibited (Fig. 3f). These results demonstrated that the reduced expression of ANGPTL2 inhibited tumor cell migration and invasion.

To clarify the biological effects observed is not the cell line specific, we obtained another PTC cell line BCPAP. BCPAP cells were transfected with an expression construct carrying ANGPTL2 cDNA, which results in overexpression of ANGPTL2. After 48 h’ transfection, the level of ANGPTL2 was confirmed by western blotting (Fig. 3g top). ANGPTL2 over-expressed cells were cultured in 96-well plates and stained with CCK8 at 72 h. We found that overexpression of ANGPT2 promoted the proliferation in BCPAP cell (Fig. 3g bottom). The results suggested that the effect of ANGPTL2 was generalizable in PTC.

### ANGPTL2 level is positively correlated with cell proliferation and migration/invasion in thyroid cancer

**Fig. 4:**
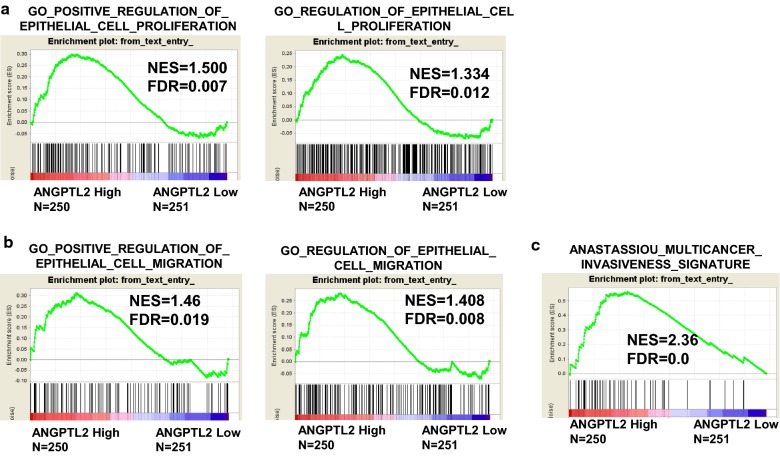
ANGPTL2 level is positively correlated with cell proliferation and migration/invasion in thyroid cancer. **a** Gene signatures for cell proliferation (GO_POSITIVE_REGULATION_OF_EPITHELIAL_CELL_PROLIFERATION and GO_REGULATION_OF_EPITHELIAL_CELL_PROLIFERATION) were enriched in a subgroup with higher ANGPTL2 expression. **b** The gene signature for cell migration (GO_POSITIVE_REGULATION_OF_EPITHELIAL_CELL_MIGRATION and GO_REGULATION_OF_EPITHELIAL_CELL_MIGRATION) was enriched in a subgroup with higher ANGPTL2 expression. **c** The gene signature for cell invasion (ANASTASSIOU_MULTICANCER_INVASIVENESS_SIGNATURE) was enriched in a subgroup with higher ANGPTL2 expression. Data were obtained from the TCGA dataset. False discovery rate (FDR) provides the estimated probability that a gene set with a given normalized ES (NES) represents a false-positive finding; FDR < 0.25 is an accepted cutoff for the identification of biologically significant gene sets

To further investigate correlations between mRNA levels of ANGPTL2 and proliferative or migration/invasion signals in thyroid cancer, data from the TCGA dataset were divided into high/low ANGPTL2 expression groups according to median and assessed by gene set enrichment analysis (GSEA). The results indicated that the gene signatures for proliferation and migration/invasion were enriched in patients with high levels of ANGPTL2 (Fig. 4a–c).

**Fig. 5:**
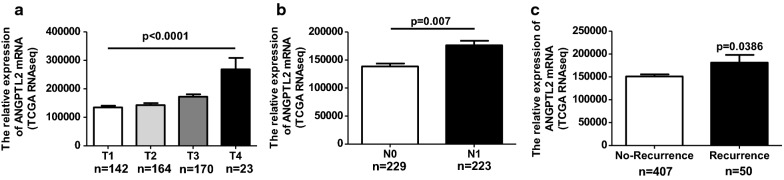
The mRNA level of ANGPTL2 increased in advanced T stage, N stage and recurrence patients. **a** ANGPTL2 mRNA increased with an increase in the T stage of thyroid cancer, (p < 0.05). **b** ANGPTL2 mRNA level at the N0 stage of thyroid cancer was significantly lower than at the N1 stage, (p < 0.05). **c** ANGPTL2 mRNA level in thyroid cancer patients with or without recurrence (p < 0.05). Data were obtained from the TCGA dataset

To further verify whether the mRNA levels of ANGPTL2 are related to thyroid cancer cell proliferation and decreased in thyroid cancer migration/invasion in clinical specimens, we analyzed the mRNA levels of ANGPTL2 in TCGA data base according to TNM stage. We found ANGPTL2 mRNA level was positively correlated with the T stage (Fig. 5a), which reflected the tumor size and invasion. Next, the ANGPTL2 mRNA levels were compared between N0 and N1 stage. The result revealed that the ANGPTL2 mRNA level was upregulated in the N1 stage (reflecting a lymphatic metastasis situation) comparing to the N0 (no lymphatic metastasis) stage (Fig. 5b), Moreover, the association of ANGPTL2 mRNA level with recurrence was assessed with the TCGA dataset as well. The result showed that patients with recurrence had a significantly increased ANGPTL2 mRNA level comparing to those without recurrence (Fig. 5c). These data indicated that the mRNA levels of ANGPTL2 may contribute closely to thyroid cancer development, and correlated to cancer cell proliferation and migration/invasion.

### High level of ANGPTL2 predicts poor clinical outcome in patients with thyroid cancer

**Fig. 6:**
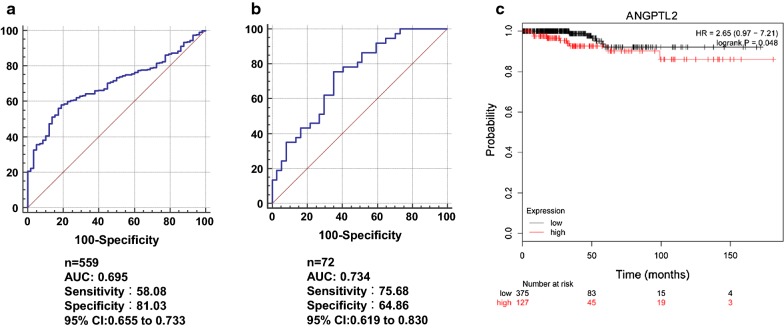
High level of ANGPTL2 predicts poor clinical outcome in patients with thyroid cancer. **a** ROC analysis for evaluation of the accuracy of ANGPTL2 mRNA level for discrimination of thyroid cancer from the TCGA dataset. **b** ROC analysis of ANGPTL2 protein levels in thyroid cancer by immunohistochemical staining. **c** Kaplan–Meier curves for overall survival of patients with thyroid cancer

Furthermore, the utility of ANGPTL2 as a predictive biomarker for thyroid cancer was evaluated by receiver operating characteristic (ROC) analysis with the data from TCGA (Fig. 6a, b). The area under the ROC curve (AUC) for ANGPTL2 mRNA was 0.695. The area under the ROC curve for ANGPTL2 protein was 0.734. These data suggested that ANGPTL2 expression level could be a predictor for diagnosis of thyroid cancer.

Online survival analysis using the thyroid cancer database in K-M Plotter demonstrated that the survival time for patients with high ANGPTL2 mRNA level were significantly less than the patients with low ANGPTL2 mRNA level [log-rank test, p < 0.05; hazard ratio (HR), 2.65; 95% confidence interval (CI), 0.97–7.21] (Fig. 6c). Taking together, the level of ANGPTL2 may serve as a prognostic indicator for patients with thyroid cancer.

## Discussion

Although the prognosis for most thyroid cancer patients is good [22], still some patients with PTC have capsular invasion to lymph node (incidence of 5%–20%) and distant metastasis, as well as incidence of disease recurrence, which results in a higher rate of mortality [23, 24]. In addition, tumorigenesis involves changes in complex molecular networks. Therefore, searching for new potential molecular markers and elucidating their molecular mechanisms in the development of thyroid cancer become necessary. It is reported that obesity is positively associated with the risk of thyroid cancer [14]. ANGPTL2 is abundantly secreted by adipose tissue. It correlated to the degree of adiposity and inflammation [18]. In addition, abnormal expression of ANGPTL2 in various malignant tumors suggested the involvement of ANGPTL2 in cancer cell proliferation, metastasis and invasion [15, 17, 20, 25, 26]. Nevertheless, lacking the studies on the correlation between ANGPTL2 expression level, and proliferative/invasive ability in thyroid cancer, leave a gap for further deep investigation about the possibility of ANGPTL2 being as potential molecular target therapeutically. Hence, our purpose in the present study was to explore the difference in expression level of ANGPTL2 between PTC and normal thyroid or precancerous tissue, as well as its impact on PTC cell proliferation, metastasis and invasion, thereby trying to investigate its possibility to become a novel target for better PTC therapy and prognosis.

This study evaluated 36 patients with PTC and found the protein level of ANGPTL2 was higher in thyroid cancer tissue than adjacent normal thyroid tissue. The mRNA Level of ANGPTL2 increased with cancer severity stage and positively correlated with tumor volume. These results suggested that ANGPTL2 accelerated PTC progression and tumor growth. Several research evidences have showed that ANGPTL2 was upregulated in various human tumor tissues and correlated with tumor size and severity [22–24]. ANGPTL2 expression has been correlated with the frequency of carcinogenesis in chemically-induced skin squamous-cell carcinoma of mice [20]. Furthermore, ANGPTL2 has been shown to promote the genesis and development of pulmonary cancer cells [14] as well as gastric cancer by accelerating the incidence and progression of disease [18]. All these studies agree with our finding. In contrast, Kikuchi et al. reported ANGPTL2 to be a putative tumor suppressor in ovarian cancer [25]. This may suggested that ANGPTL2 could act differently in different cancer. But overall, all research data supported that ANGPTL2 may act as a critical factor in cancer development.

In the study, we found that ANGPTL2 overexpression in TPC-1 cells increased cellular proliferation. ANGPTL2 Knockdown with small interfering RNA decreased cellular proliferation and inhibited tumor cell migration. Further, gene signatures for proliferation were enriched in patients with higher levels of ANGPTL2 compared to those with lower levels of ANGPTL2.

ANGPTL2 downregulation by siRNA has been shown to inhibit cell growth, migration, and invasion of prostate cancer cells (LNCaP) [17]. High level of ANGPTL2 expression has also been shown to accelerate pulmonary cancer cell invasion and metastasis through autocrine and paracrine mechanisms [15, 20], to drive metastasis of human lung cancer [15, 22], osteosarcoma cell lines [26], and breast cancer [27]. The effect of ANGPTL2 in induction of tumor cell metastasis via different protein in different type of cancer have been reported, such as integrin α5β1, p38 mitogen activated protein kinase (MAPK), and matrix metalloproteinase (MMP)-9 [26, 28].

The PI3K/AKT and ERK/MAPK signaling pathway are important signal transduction pathways in gene expression and cellular functions. Aberrant activation of the PI3K/AKT and ERK/MAPK signaling pathway are often found in tumors including thyroid cancer [29]. It was reported that the genes, coding the MAPK/ERK and PI3K/AKT signaling cascade proteins (e.g., RET, RAS, BRAF, PI3K, PTEN, AKT), were significantly mutated in thyroid cancer, which contributed to aberrant activations of MAPK/ERK and PI3K/AKT signaling pathways of thyroid cancer [30]. Recently, many studies have proven that aberrant signaling through the RAS/RAF/MEK/ERK cascade is a crucial factor for thyroid tumour initiation and development [31]. Clinical correlations and experimental in vitro and in vivo data demonstrated that PI3K/AKT signaling pathway played the key regulatory role, in the formation and progression of thyroid tumor. Hou et al. reported that 24% of PTCs and 31% of benign follicular adenomas contained one of PI3K abnormalities [32]. Therefore, we tested the expression of pERK1/2 and pAKT in adjacent tissue and thyroid tumor tissue by IHC. The data showed the level of pERK1/2 and pAKT increased in thyroid tumor tissue compared with adjacent normal thyroid tissue in 36 patients with PTC (Additional file 1: Fig. S2A, B). We further investigated the association of ANGPTL2 expression and ERK1/2 and AKT activation gene signature by GSEA, and found the gene signatures of ERK1/2 and AKT activation were both enriched in high ANGPTL2 expression group (Additional file 1: Fig. S2C, D). These results suggested that ANGPTL2 promoting thyroid cancer cell function maybe mediated by ERK1/2 and AKT signaling pathways.

The current study has potential limitations. First, a clear elucidation of the cell signaling pathways involved in proliferation, metastasis, and invasion of PTC cells will be required for further detail studies. Second, we did not determine circulating plasma levels of ANGPTL2 protein in patients with PTC or in non-cancer participants.

## Conclusion

The key findings of this study were: (1) ANGPTL2 expression was higher in thyroid cancer cell in comparison to adjacent normal thyroid tissue cell; (2) ANGPTL2 expression was increased with thyroid tumor progression; (3) high expression of ANGPTL2 enhanced proliferation of thyroid cancer cells; (4) ANGPTL2 promoted cell migration and invasion of thyroid cancer cells; (5) higher level of ANGPTL2 in thyroid cancer patients were significantly associated with a poor prognosis. In summary, this study demonstrated that ANGPTL2 participated in PTC cell proliferation, metastasis, and invasion, which suggested the possibility of ANGPTL2 being as a new therapeutic target for treatment of PTC patients.

## Additional file

**Additional file 1.** **Fig. S1.** ANGPLT2 promote cell cycle at both G1/S and G2/M checkpoint in TPC-1 cells. A. ANGPTL2overexpression led to G1/S and G2/M phase accumulation in TPC-1 cells. Cell transfected with HA-vector or HA-ANGPTL2 and cell cycle analysis was performed using propidium iodide DNA staining and flow cytometry. B, C. The gene signatures of cell cycle (BIOCARTA_G1_PATHWAY and BIOCARTA_G2_PATHWAY) were enriched in patients with high ANGPTL2 expression. **Fig. S2.** ANGPTL4 level was positively correlated with the activation of ERK1/2 and AKT. A, B. The protein levels pERK1/2 and pAKT of 36 human thyroid cancers and adjacent tissues were analyzed by immunohistochemistry. C, D. Gene signatures of ERK1/2 and AKT activation were enriched in patients with high ANGPTL2 expression, and data were from TCGA datasets.

## Data Availability

Not applicable.

## Abbreviations

PTC: papillary thyroid carcinoma
ANGPTL2: angiopoietin-like 2
CCK-8: cell counting kit-8
GCEA: gene set enrichment analysis
ROC: receiver operating characteristic
HR: hazard ratio
CI: confidence interval
MAPK: mitogen activated protein kinase
MMP-9: matrix metalloproteinase 9
