# Supplementary material for: Expression of ANGPTL2 and its impact on papillary thyroid cancer
Source: Cancer Cell Int. 2019 Jul 30;19:204. doi: 10.1186/s12935-019-0908-9 (PMC6668118; doi:10.1186/s12935-019-0908-9)

Additional file 1: Figure S1

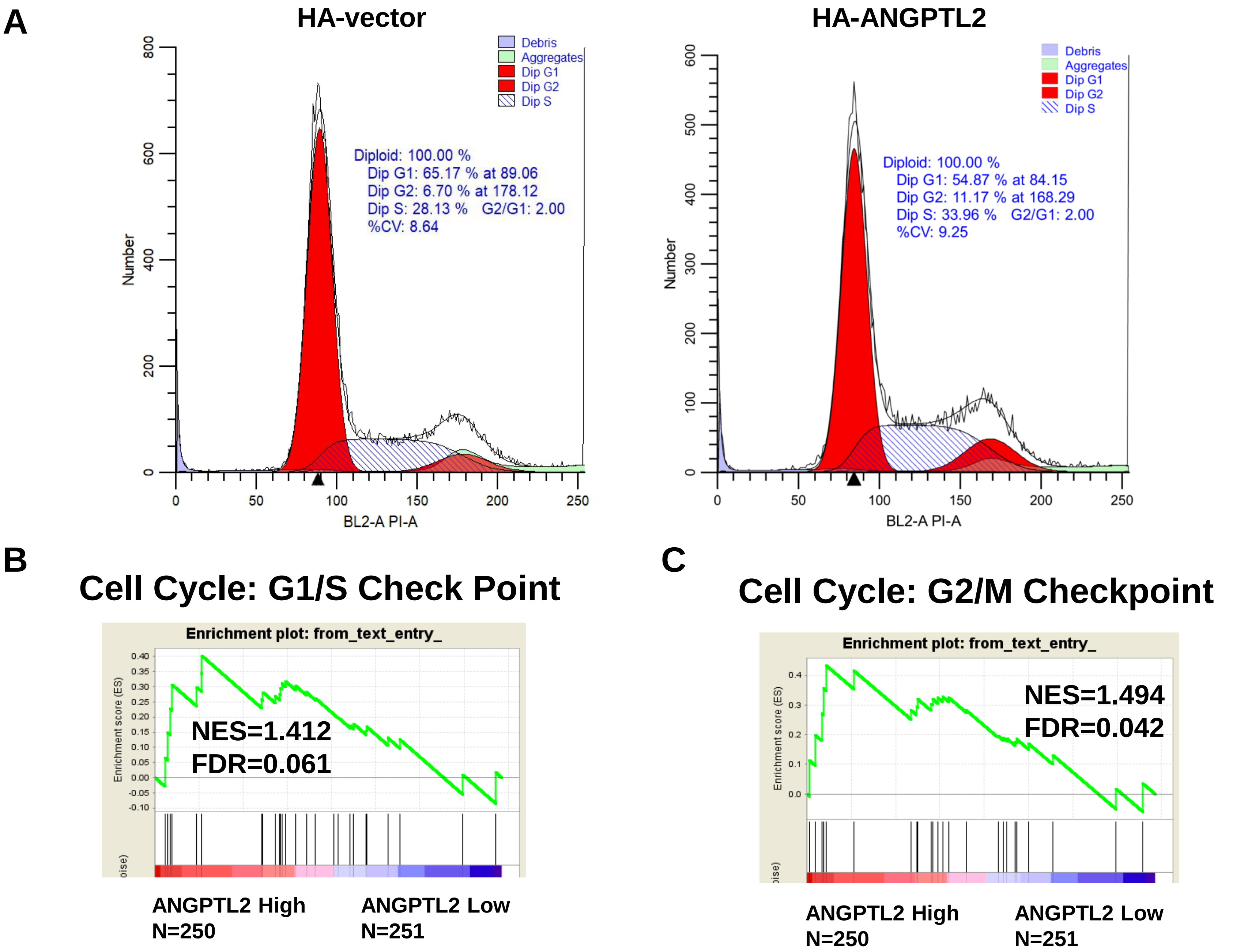

Additional file 1: Figure S2

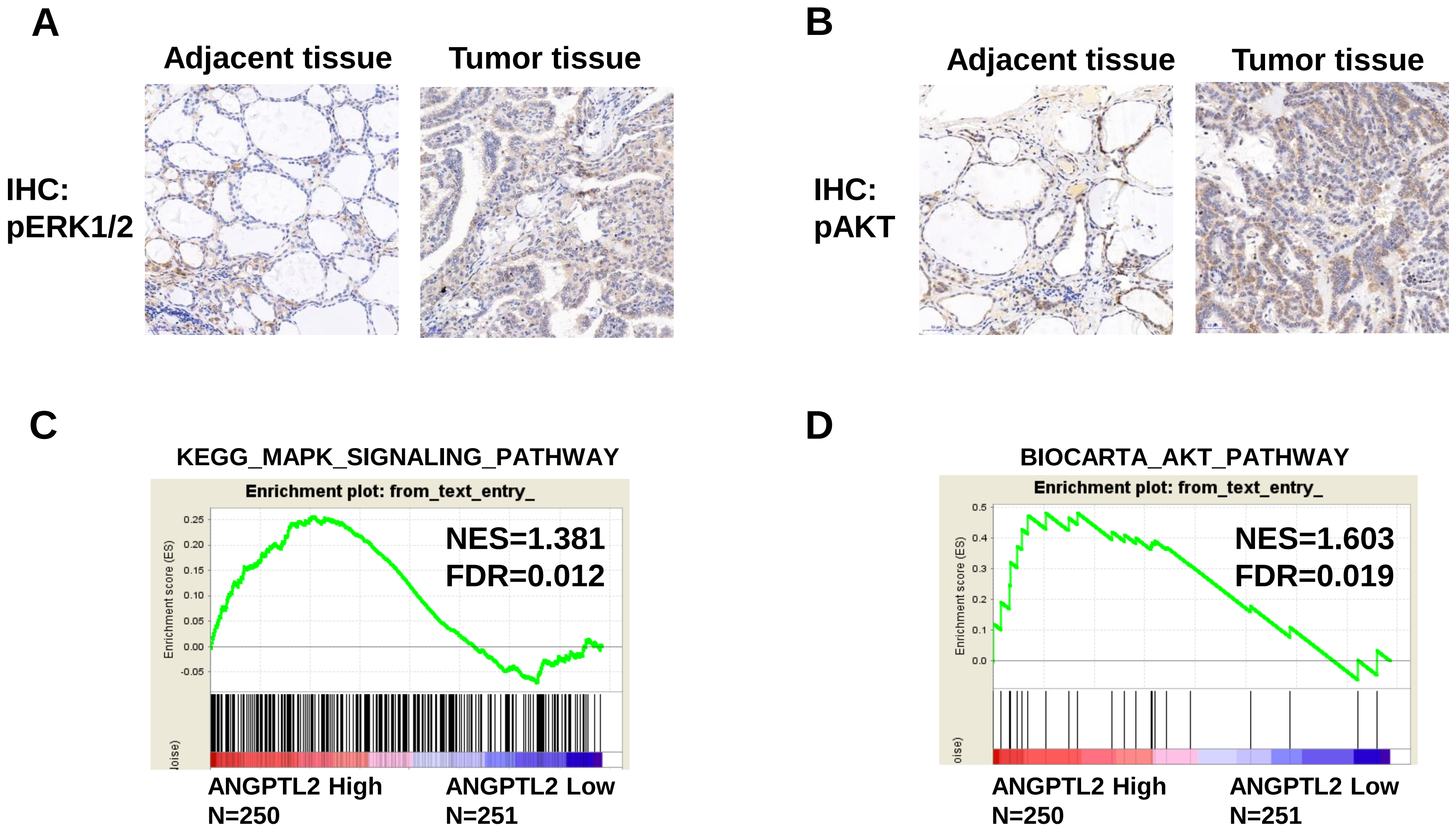

Supplement: Supplementary file 1 — Additional file 1. Fig. S1. ANGPLT2 promote cell cycle at both G1/S and G2/M checkpoint in TPC-1 cells. A. ANGPTL2overexpression led to G1/S and G2/M phase accumulation in TPC-1 cells. Cell transfected with HA-vector or HA-ANGPTL2 and cell cycle analysis was performed using propidium iodide DNA staining and flow cytometry. B, C. The gene signatures of cell cycle (BIOCARTA_G1_PATHWAY and BIOCARTA_G2_PATHWAY) were enriched in patients with high ANGPTL2 expression. Fig. S2. ANGPTL4 level was positively correlated with the activation of ERK1/2 and AKT. A, B. The protein levels pERK1/2 and pAKT of 36 human thyroid cancers and adjacent tissues were analyzed by immunohistochemistry. C, D. Gene signatures of ERK1/2 and AKT activation were enriched in patients with high ANGPTL2 expression, and data were from TCGA datasets. [file 12935_2019_908_MOESM1_ESM.pdf]
